# Unconventional Photoluminescence in Tin Iodide Perovskite Nanocrystals: A Perspective

**DOI:** 10.1021/acs.jpclett.6c00096

**Published:** 2026-02-17

**Authors:** Sumit Kumar Dutta, Jia-Kai Chen, Naoto Shirahata, Hong-Tao Sun

**Affiliations:** † Research Center for Materials Nanoarchitectonics (MANA), National Institute for Materials Science (NIMS), Tsukuba 305-0047, Japan; ‡ Graduate School of Chemical Sciences and Engineering, Hokkaido University, Sapporo 060-0814, Japan; § Department of Physics, Chuo University, Tokyo 112-8551, Japan

## Abstract

Tin iodide perovskite nanocrystals are compelling lead-free candidates for solution-processed optoelectronics, yet their reported photoluminescence (PL) signatures are marked by persistent and unresolved anomalies. Literature reports show that PL energies can vary widely among nanocrystals of comparable size and that charge carriers can exhibit decoupling between the PL quantum yield and PL lifetime, along with slow hot-carrier relaxation dynamics. Low-temperature studies introduce further complexity, including the emergence of additional emissive features and nonmonotonic spectral evolution. In this Perspective, we consolidate these seemingly disparate observations into a unified framework and critically assess the key factors that complicate the interpretation of tin iodide nanocrystal photophysics. These include polymorphous or locally distorted crystal structures, structural defects coupled with hole doping, and trace two-dimensional Ruddlesden–Popper phases that can dominate the observed PL while evading routine structural characterization. Finally, we outline actionable research directions, such as the phase-pure synthesis of highly luminescent nanocrystals through rational ligand and precursor control or doping engineering, defect-tolerant surface design, and stringent structure–spectroscopy correlations, to transform apparent “anomalies” into testable physical mechanisms and establish robust structure–photophysics relationships for tin halide perovskite nanocrystals.

## Graphical abstract

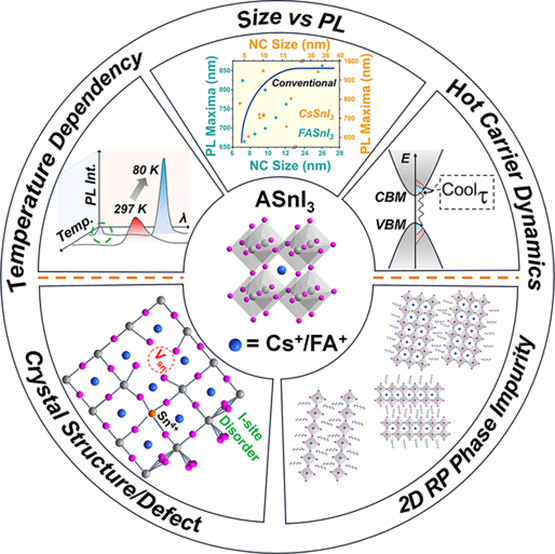

Colloidal lead halide perovskite nanocrystals (NCs) have set benchmarks in solution-processed optoelectronics owing to their high photoluminescence quantum yields (PLQYs), narrow emission line widths, large absorption coefficients, and broadly tunable bandgaps.^1–5^ These attributes have enabled record-level performance in light-emitting diodes and photovoltaic devices.^6–8^ Nevertheless, the intrinsic toxicity and high bioavailability of lead remain major barriers to their widespread practical implementation.^9,10^ These concerns have prompted the research community to search for biocompatible alternatives, among which tin halide perovskites have emerged as the most chemically analogous substitutes, as Sn^2+^ closely resembles Pb^2+^ in both electronic configuration and ionic radius.^11–13^ Within this material class, tin iodide perovskites (ASnI_3_, where A is Cs^+^, formamidinium (FA^+^), or methylammonium (MA^+^)) are particularly attractive, as they feature direct bandgaps of 1.2–1.4 eV, high carrier mobility, long carrier diffusion lengths, and photovoltaic efficiencies exceeding 14%.^14–18^

Recent years have witnessed remarkable progress in ASnI_3_ perovskite NCs, encompassing advances in synthetic methodologies, defect chemistry, and surface engineering.^14^ Since the first colloidal synthesis of cesium tin iodide (CsSnI_3_) NCs via a hot-injection route reported by Jellicoe et al. in 2016,^16^ extensive efforts have been devoted to exploring the synthesis of both CsSnI_3_ and organic–inorganic hybrid FASnI_3_ NCs.^14,19–30^ Parallel progress in elucidating the defect chemistry of ASnI_3_ perovskite NCs has revealed that suppressing intrinsic Sn vacancies and constructing defect-tolerant surfaces are critical for enhancing their optical performance.^19–21^ Moreover, several ligand engineering strategies have also been developed to enhance the stability of ASnI_3_ perovskite NCs, thereby broadening their prospects for optoelectronic applications.^25,31^

Despite these advances in the synthetic chemistry of ASnI_3_ NCs, several intriguing yet perplexing observations regarding the photoluminescence (PL) properties have emerged in the literature. Notably, pronounced bandgap widening relative to bulk materials is frequently observed even when NC sizes exceed the calculated exciton Bohr diameters,^23,26,32^ deviating markedly from the quantum confinement effect established for classical semiconductor NCs.^33–35^ In addition, anomalous carrier dynamics and low-temperature PL properties have also been reported.^28,36^ These anomalous PL characteristics have received limited attention, and their underlying mechanisms remain largely unresolved. Given the considerable promise of ASnI_3_ NCs for the next-generation, environmentally benign optoelectronic devices, a critical consolidation and reassessment of these phenomena is timely, as a clearer understanding of their optical behaviors is expected to inform future synthetic design, refine mechanistic interpretations, and stimulate more rigorous investigations aimed at resolving these outstanding ambiguities.

In this Perspective, we summarize the unconventional PL characteristics reported for ASnI_3_ NCs (A = Cs^+^ or FA^+^), encompassing steady-state and time-resolved PL as well as low-temperature photophysical properties. Particular emphasis is placed on the roles of crystal structure, defects, hole doping, and trace impurities in affecting their optical behaviors. Finally, we outline potential strategies to bridge current synthetic and mechanistic gaps, with the aim of unlocking the full photophysical potential of this emerging class of perovskite nanomaterials.

## Size–Luminescence Anomalies

We began by examining size-dependent luminescence behaviors in ASnI_3_ NCs, where some of the most striking and widely reported PL anomalies first emerge. The earliest colloidal CsSnI_3_ nanocubes, synthesized by Jellicoe et al., have a lateral dimension of ∼10 nm and exhibit a PL maximum near 950 nm.^16^ Since then, multiple studies employing similar hot-injection strategies have produced CsSnI_3_ NCs within a similar size regime; however, these NCs display markedly different optical characteristics, often exhibiting substantial blueshifts to shorter wavelengths.^23,31,32^ For instance, Kang et al. synthesized CsSnI_3_ NCs with an average size of 10.4 nm, which display a PL maximum of 849 nm,^31^ whereas Gahlot et al. and Li et al. observed PL maxima near 700 nm from NCs of comparable size.^23,32^ Most reported CsSnI_3_ NCs crystallize in the orthorhombic phase, whereas those studied by Li et al. were assigned to the cubic phase.^32^ Theoretical calculations indicated that cubic-phase CsSnI_3_ possesses a direct bandgap that is slightly smaller than that of its orthorhombic counterpart.^37–39^ Consequently, the pronounced PL blueshift observed in the NCs reported by Li et al. cannot be rationalized solely by the change in crystal symmetry.^32^ Size-dependent discrepancies become even more pronounced in smaller nanostructures. For example, CsSnI_3_ NCs with an average size of ∼6 nm exhibit band-edge absorption near 590 nm and PL centered at ∼606 nm,^23^ whereas nanoplatelets with a thickness of ∼3.8 nm, synthesized using mixed amine ligands, display much longer-wavelength emission near 780 nm.^20^ If quantum confinement was the dominant factor governing the PL of the ∼6 nm CsSnI_3_ NCs, such a PL blueshift would be difficult to reconcile with the reported emission characteristics of the thinner nanoplatelets.

Similar ambiguities persist in the size-dependent PL behaviors of FASnI_3_ NCs. For instance, Dai et al. reported a size-tunable synthesis achieved by controlling the growth temperature, yielding FASnI_3_ NCs with sizes ranging from 7.3 to 12.1 nm with PL peaking between 666 and 763 nm.^28^ The exciton Bohr diameter of cubic-phase FASnI_3_ with a bandgap of 1.41 eV was estimated to be approximately 8.8 nm.^29^ Accordingly, NCs larger than this dimension are expected to exhibit near-band-edge PL or, at most, modest blueshifts. However, 9.7 nm FASnI_3_ NCs display PL peaking shorter than 710 nm, corresponding to a blueshift exceeding 0.34 eV relative to the bulk value.^28,40^ In contrast, Chen et al. obtained ∼7.1 nm FASnI_3_ NCs that exhibit a PL maximum around 825 nm.^30^ Such pronounced discrepancies strongly suggest the involvement of additional factors beyond size alone. Notably, oleylamine was used in most of the syntheses of these NCs, which, as we found in subsequent experiments, can easily introduce two-dimensional (2D) Ruddlesden–Popper (RP) perovskite impurities. Correspondingly, we developed a zwitterionic ligand-involved synthesis that yields 2D-perovskite-free, highly luminescent FASnI_3_ NCs with sizes exceeding 20 nm.^19^ Further exploration of such synthetic routes, particularly for small-sized FASnI_3_ NCs, may provide phase-pure model systems for interrogating quantum-confinement effects and establishing more reliable size- and structure–PL relationships.

Table 1 summarizes representative literature reports on CsSnI_3_ and FASnI_3_ NCs, highlighting their morphologies, particle sizes, absorption onsets, PL peak positions, and employed synthetic methods, and collectively underscores the anomalous relationship between particle size and PL energy. To visualize this trend, reported PL maxima are plotted versus NC size using literature data (Figure 1). The bandgaps of bulk orthorhombic CsSnI_3_ and cubic FASnI_3_ perovskites are 1.30 and 1.41 eV, respectively, as established by experimental measurements and theoretical calculations.^15,40^ Notably, highly luminescent, phase-pure CsSnI_3_ (37 nm) and FASnI_3_ (26 nm) NCs exhibit emission maxima comparable to these bulk values,^19,21^ indicating bulk-like optical behaviors at sufficiently large sizes (Figure 1). In contrast, upon size reduction to below ∼18 nm, both CsSnI_3_ and FASnI_3_ NCs exhibit highly inconsistent size-dependent emission maxima across different reports. At least three distinct size–luminescence anomaly regimes can be identified for both CsSnI_3_ and FASnI_3_ NCs. In particular, the PL maxima of 9–11 nm CsSnI_3_ NCs span a broad spectral range from ∼700 to ∼950 nm, with similar variability observed for ∼10 nm FASnI_3_ NCs (green rectangular regions in Figure 1a, b). Moreover, within the blue and yellow elliptical regions in Figure 1a, b, the PL maxima do not shift monotonously toward shorter wavelengths with deceasing NC size, as conventionally expected. Collectively, these behaviors deviate markedly from the conventional size–bandgap trends observed in lead halide perovskite NCs and other semiconductor NCs.^33–35^ Such anomalous size–PL correlations are therefore unlikely to arise from quantum confinement alone and cannot be straightforwardly ascribed to the intrinsic emission of CsSnI_3_ and FASnI_3_ NCs. Instead, they likely reflect the interplay of additional, size-independent factors, including crystal structure, structural defects, p-type doping, trace 2D RP perovskite impurities, or combinations thereof, as discussed in the following sections.

**Table 1:** Summary of Literature Reports on the Morphologies, Particle Sizes, Optical Properties, and Synthesis Methods for CsSnI_3_ and FASnI_3_ NCs

ASnI_3_	Morphology	Particle size (nm)	Absorption onset (nm)	PL maxima (nm)	Ligands^b^	Reaction condition^c^	ref.
**CsSnI**_**3**_	Nanocubes	9.9 ± 3.9	∼920	∼950	OLA + OA	H–I	^16^
	Nanocubes	16 ± 5	629.1	657.6	OLA + OA	CE	^41^
	Not defined	7		938	OLA + OA	H–I	^42^
	Nanoplatelets	∼3.8^a^	∼780	∼780	OLA + OctAm + OctA	H–I	^33^
	Nanocubes	10.4 ± 1.3	∼1000	∼849	OLA + OA	H–I	^31^
	Nanocubes	37		∼944	OLA + OA	H–I	^21^
	Nanocubes	6 ± 1	590	∼606	OLA + OA	H–I	^23^
		10 ± 1	705	∼714	OLA + OA	H–I	^23^
	Nanocubes	17.2 ± 1.9	∼800	804	OLA + OA + thiourea	H–I	^25^
	Nanocubes	9 ± 1	∼690	∼703	OLA + OA	H–I	^32^
	Nanocubes	8.9 ± 1	∼713	∼721	OLA + OA	H–I	^36^
	Nanocubes	10	∼705	∼721	OLA + OA	H–I	^26^
	Nanocubes	∼10	∼702	∼712	OLA + OA	H–I	^43^
**FASnI**_**3**_	Nanocubes	7.3 ± 0.8 to 12.1 ± 1.1	∼650–730	∼666–763	OLA + OA	H–I	^28^
	Nanocubes	9.7 ± 1.1	∼800	∼800	OLA + OA	H–I	^29^
	Not defined	7.1 ± 1.8		∼825	OLA + OA	LARP	^30^
	Nanocubes	26 ± 6		860 – 867	C_44_–PC + OA	H–I	^19^

a This value is the thickness of the nanoplatelets.

b In this column, oleylamine, oleic acid, octylamine, octanoic acid, and dioleoyl-*sn*-glycero-3-phosphocholine are abbreviated as OLA, OA, OctAm, OctA, and C_44_–PC, respectively.

c In this column, H–I, CE, and LARP stand for hot injection, cation exchange, and ligand-assisted reprecipitation, respectively.

**Figure 1:**
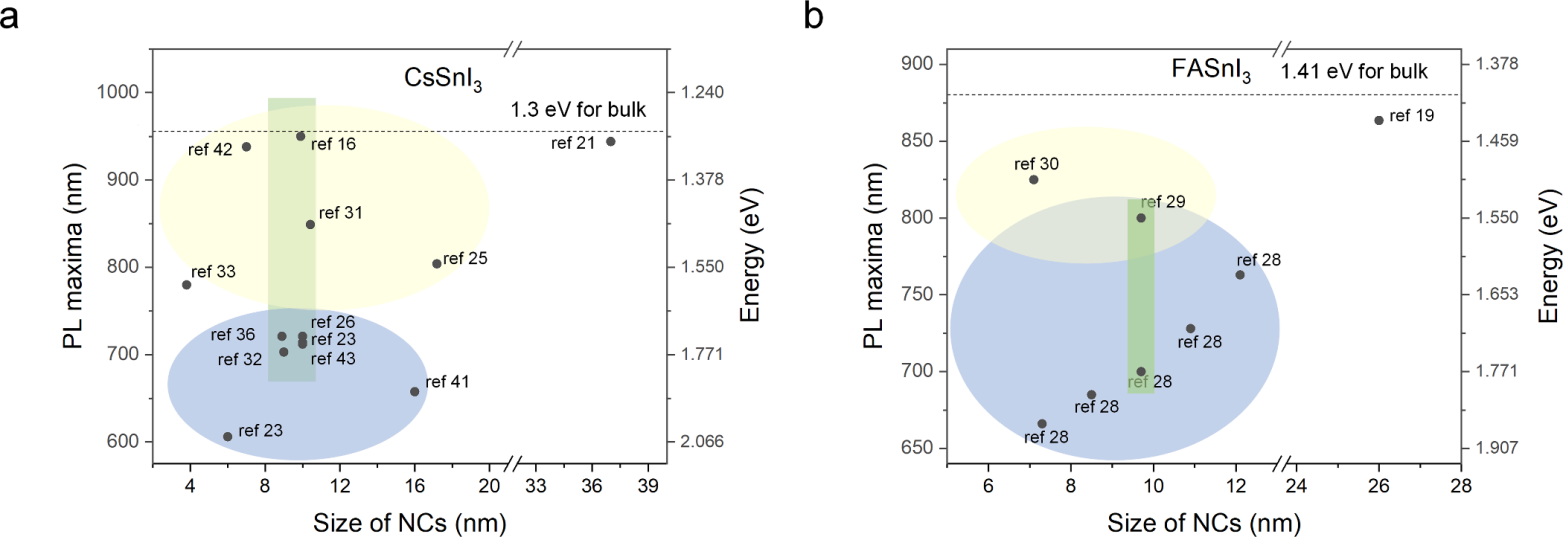
Plot of the literature survey of unconventional size-dependent PL maxima in (a) CsSnI_3_ and (b) FASnI_3_ NCs. The dotted lines indicate the bandgaps of bulk orthorhombic CsSnI_3_ and cubic FASnI_3_ perovskites. The light-yellow and blue elliptical regions denote regimes of anomalous size-dependent PL behavior, while the green rectangles highlight large variations in PL maxima among NCs with similar sizes.

## Anomalies in Photogenerated Charge-Carrier Dynamics

Beyond size-luminescence anomalies, unconventional photophysical behaviors in ASnI_3_ NCs were also manifested in their photogenerated charge-carrier dynamics. In semiconductor NCs, a higher PLQY typically reflects more effective passivation of surface and bulk defects that would otherwise introduce nonradiative recombination pathways and result in poor PLQYs.^44–46^ This correlation is well established in lead halide perovskite NCs, where enhancements in PLQY, achieved through surface passivation or doping, generally correlate with longer PL lifetimes.^44,46,47^ For tin halide perovskite NCs, similar correlations have been observed in selected cases, although notable deviations have also been reported. For example, Liu et al. reported the synthesis of luminescent colloidal CsSnI_3_ NCs through careful optimization of precursor stoichiometry.^21^ As shown in Figure 2a, the PL decay dynamics of CsSnI_3_ NCs vary systematically with the Cs:Sn ratio: the average lifetime increases from 0.85 ns (0.25:3) to 1.36 ns (0.25:4.8), in parallel with an increase in PLQY from 4.3% to 18.4%. Based on the measured PLQYs and PL lifetimes, the corresponding radiative and nonradiative recombination rates were estimated. Notably, increasing the Sn content reduces the nonradiative recombination rate by more than a factor of 2, from 1404.47 μs^–1^ to 600.22 μs^–1^ (Figure 2b). These results clearly indicate that judicious tuning of constituent chemical potentials can suppress nonradiative recombination, therefore prolonging PL lifetimes and enhancing PLQYs.

**Figure 2:**
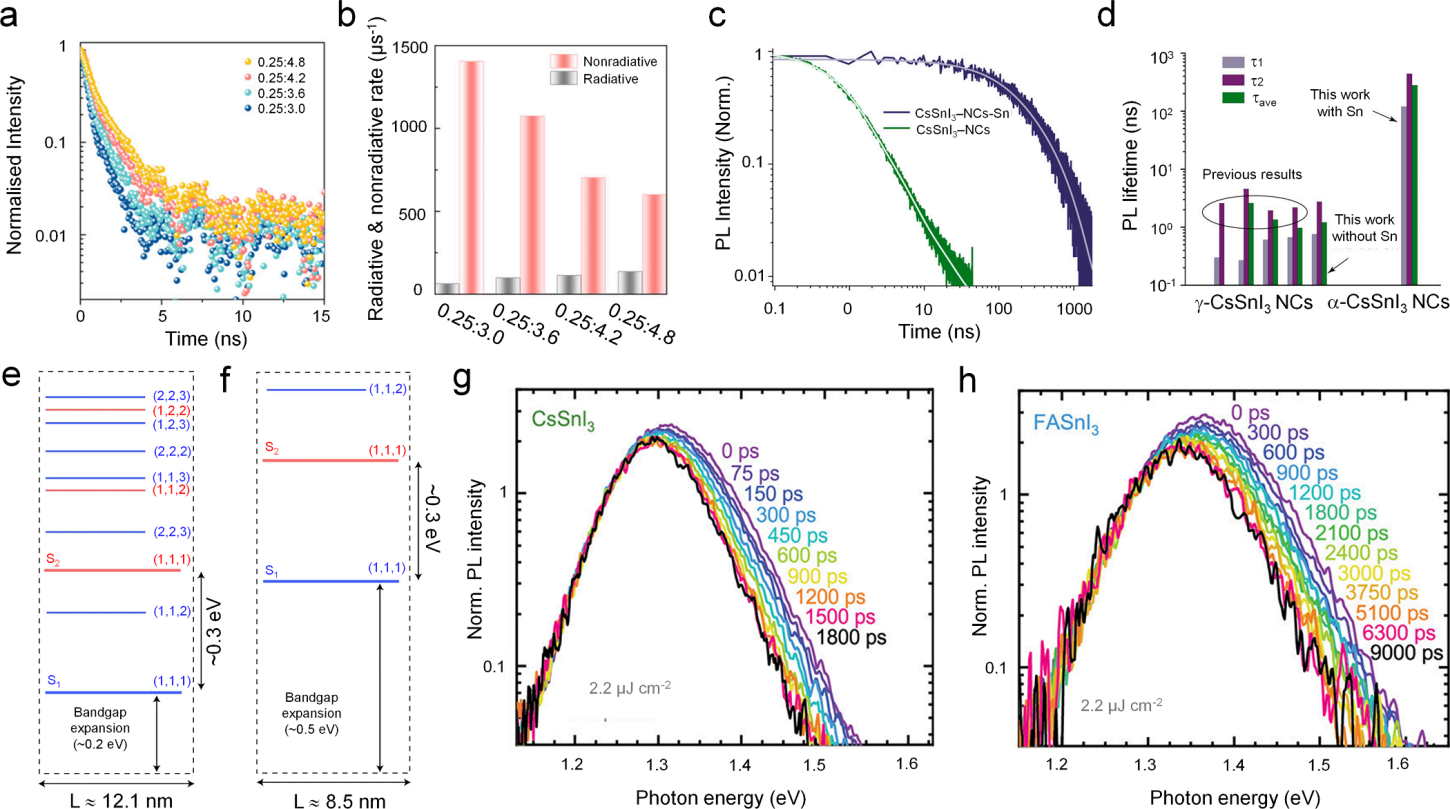
(a) PL decay traces and (b) histograms of the radiative and nonradiative recombination rates for CsSnI_3_ NCs with different Cs:Sn precursor ratios. Reproduced with permission from ref 21. Copyright 2021 American Chemical Society. (c) Normalized PL kinetics of CsSnI_3_ NCs-Sn and CsSnI_3_ NCs. (d) Lifetime statistics for CsSnI_3_ NCs. τ_1_, τ_2_, and τ_ave_ correspond to individual lifetimes of two decay components and the calculated average decay lifetime, respectively. Reproduced with permission from ref 32. Copyright 2024 American Chemical Society. (e,f) Quantized energy levels in ∼12.1 nm and ∼8.5 nm FASnI_3_ NCs, respectively, assuming an ideal quantum box model. Reproduced with permission from ref 28. Copyright 2021 Nature Publishing Group. (g, h) Energy-dependent PL spectra at indicated times after initial 2.2 μJ cm^–2^ laser pulse excitation, normalized at the red tail for CsSnI_3_ and FASnI_3_ respectively. Reproduced with permission from ref 51. Copyright 2024 American Chemical Society.

Recently, Li et al. reported the synthesis of α-phase CsSnI_3_ NCs using a solid–liquid antioxidation suspension composed of tri-n-octylphosphine (TOP) and zerovalent tin (Sn(0)).^32^ Upon Sn(0) treatment, CsSnI_3_ NCs exhibit an increase in PLQY from ∼0.1% to ∼6.3%, yet display an extraordinary extension of the PL lifetime, from 1.22 to 278.30 ns. Concurrently, the crystal structure was reported to transform from the orthorhombic phase to the cubic phase after Sn(0) treatment. Figure 2c compares the PL decay of the Sn(0)-treated NCs (denoted as CsSnI_3_ NCs-Sn) with that of untreated control NCs (denoted as CsSnI_3_ NCs). As summarized in Figure 2d, the markedly prolonged lifetime correlates with a strong suppression of nonradiative recombination, indicating that Sn(0) treatment effectively mitigates certain PL-detrimental defects. It is worth noting that these ∼9 nm NCs exhibit a pronounced PL blueshift (∼0.46 eV) relative to bulk CsSnI_3_. Furthermore, despite their ultralong PL lifetimes, the PLQYs remain substantially lower than those of CsSnI_3_ NCs reported by Liu et al.^21^ This unusual combination of strong PL blueshift, ultralong lifetime, and moderate PLQY raises important mechanistic questions. Several possible scenarios may account for these observations. First, the PL blueshift could arise from strong quantum confinement effects, and the extended lifetime might originate from phase changes, provided that the emission indeed arises from cubic CsSnI_3_ NCs rather than from secondary phases. However, this interpretation remains controversial when comparing the studies by Li et al. and Jellicoe et al.,^16,32^ since crystals with slightly different structures (e.g., cubic and orthorhombic CsSnI_3_ phases, both possessing direct bandgaps) are expected to exhibit similar exciton Bohr diameters.^37^ Accordingly, further evidence is required to unambiguously assign the observed PL and ultralong lifetimes to intrinsic cubic CsSnI_3_ NCs. Second, the synthesis protocol used for Sn(0)-treated NCs may facilitate the formation of 2D RP perovskites, which could coexist with the final NC products and substantially influence carrier relaxation dynamics. As discussed below, given the spectral similarity between the observed PL and those of known 2D RP tin iodide perovskites, the emission could plausibly originate from trace RP impurities, which are often difficult to detect using conventional transmission electron microscopy (TEM) and X-ray diffraction (XRD) analyses. Third, trapping–detrapping processes associated with shallow defect states can substantially prolong PL lifetimes even in the presence of significant nonradiative recombination, and may therefore also contribute to the anomalously long PL lifetimes observed.^48,49^ Beyond these cases, additional reports documenting disparate combinations of PLQYs and PL lifetimes are summarized in Table 2. Collectively, these observations point to an anomalous decoupling between PLQY and PL lifetime in ASnI_3_ NCs, which contrasts sharply with trends established in lead halide perovskites and conventional semiconductors. Although the microscopic origins of this decoupling, as well as the stabilization mechanism of cubic CsSnI_3_ NCs at room temperature, remain unresolved, these results underscore that access to high-quality, phase-pure, and size-tunable NCs is a prerequisite for disentangling intrinsic carrier dynamics from extrinsic effects.

**Table 2:** Summary of Literature Reports on the PL Positions, PLQYs, and Corresponding PL Lifetimes of CsSnI_3_ and FASnI_3_ NCs

			PL lifetime			
ASnI_3_	PL maxima (nm)	PLQY (%)	Slow component (ns)	Fast component (ns)	Avg. (ns)	ref.
**CsSnI**_**3**_	∼950	0.06	2.65	0.3		^16^
	∼780	<1	5.65	0.72		^20^
	849	0.35	4.59	0.27	2.62	^31^
	944	18.4	1.95	0.61	1.36	^21^
	∼606	1–5%	2.08	0.43		^23^
	∼714		2.20	0.67		^23^
	∼703	6.3	437.45	118.63	278.30	^32^
	∼804	0.48	10.39	0.71	2.46	^25^
**FASnI**_**3**_	666–763	∼0.3	3.04 – 5.05	0.36 – 0.97	2.08–3.80	^28^
	∼800	0.1	1.9	0.3	1.25–1.15	^29^
	860–867	0.9–36.9	0.49–215.18	0.28–45.37	0.38–132.5	^19^

In addition to band-edge recombination dynamics, ASnI_3_ NCs also exhibit slow hot-carrier relaxation behaviors. Transient absorption measurements on FASnI_3_ NCs revealed a transition from a quasi-continuous band structure to discretized energy levels as the NCs dimension varied, which markedly slows hot-carrier cooling.^28^ The decay kinetics at the higher-energy excited states progressively slow with decreasing NC size, reflecting increasingly hindered interstate relaxation. This effect has been rationalized by the presence of two conduction-band manifolds with nearly identical effective masses, which therefore undergo similar quantum-confinement-induced energy shifts as NC size decreases (Figures 2e,f). As a consequence, the energy separation between these quantized states remains nearly constant, giving rise to a phonon bottleneck that substantially retards hot-carrier relaxation. This behavior differs strikingly from that of lead halide perovskite NCs, where CsPbBr_3_, MAPbBr_3_, and FAPbBr_3_ exhibit ultrafast subpicosecond hot-carrier cooling (0.18–0.31 ps) under identical excitation conditions, whereas FASnI_3_ NCs display a markedly prolonged cooling time of ∼15 ps. Nevertheless, it should be emphasized the investigated FASnI_3_ NCs exhibit extremely low PLQYs (<1%), indicative of a high density of defects, and may also contain trace 2D RP impurities due to the use of oleylamine ligands. Both defect states and secondary 2D phases strongly influence hot-carrier relaxation and should therefore be carefully considered.^50^

In parallel, the group led by M. A. Loi systematically investigated hot-carrier relaxation in FASnI_3_, MASnI_3_, and CsSnI_3_ films and observed nanosecond-scale hot-carrier relaxation extracted from hot-carrier PL spectra (Figure 2g and 2h).^51^ Notably, they demonstrated a pronounced high-energy shift of the emission peak with increasing excited-state population, an effect more prominent in FASnI_3_ and MASnI_3_ than in CsSnI_3_. Similar trends, namely, slower carrier cooling in hybrid perovskites relative to fully inorganic counterparts, have also been reported in lead halide perovskites.^52,53^ Moreover, the fluence-dependent PL blueshift scales linearly with the photocarrier density raised to the two-thirds power (n^2/3^), consistent with a dynamic Burstein–Moss effect.^51^

Taken together, these results highlight that ASnI_3_ NCs and their bulk or polycrystalline counterparts can exhibit fundamentally different hot-carrier relaxation pathways. Accordingly, systematic investigations of hot-carrier dynamics in highly luminescent, phase-pure, and size-tunable ASnI_3_ NCs are essential for bridging this knowledge gap and for establishing a rigorous understanding of their intrinsic carrier-relaxation behaviors.

## Anomalies in Temperature-Dependent PL Behaviors

In addition to anomalies in size-luminescence relationships and charge-carrier dynamics, temperature-dependent PL provides another critical dimension for probing unconventional photophysics in ASnI_3_ NCs. Previous studies have shown that the bandgap of CsSnI_3_ perovskite monotonically increases with increasing temperature, a trend opposite to that observed in most conventional semiconductors. This behavior has been experimentally established in CsSnI_3_ polycrystalline films and microplates, where the PL maxima shift from longer wavelengths at low temperature to about 950 nm (1.3 eV) at room temperature.^54,55^ Electronic structure calculations have revealed that CsSnI_3_ perovskite possesses a band structure distinct from most traditional semiconductors, featuring a nondegenerate *s*-like valence band maximum composed of Sn *s* and I *p* states and a triply degenerate *p*-like conduction band minimum composed predominantly of Sn *p* states. Its bandgap is essentially controlled by the Sn *s* to I *p* antibonding interaction.^37^ With an increasing lattice constant, which can be thermally induced by increasing temperature, this antibonding interaction is expected to weaken, leading to a reduction in valence-bandwidth and a concomitant increase in the bandgap. This framework provides a consistent microscopic explanation for the experimentally observed monotonic PL blueshift with increasing temperature in bulk CsSnI_3_ systems.

By contrast, the temperature-dependent PL of CsSnI_3_ NCs is considerably more complex than that of large-sized CsSnI_3_ perovskites and lead halide analogues.^56,57^ In a recent study by Kluherz et al., the excitonic emission of CsSnI_3_ NCs does not evolve via a simple linear redshift upon cooling.^36^ Instead, as shown in Figure 3a, a second, higher-energy emission band emerges below ∼240 K and persists alongside the primary excitonic PL over a broad temperature window. This behavior is inconsistent with the monotonic temperature-dependent evolution observed in lead halide analogues and was attributed to a modification of the underlying lattice potential.

**Figure 3:**
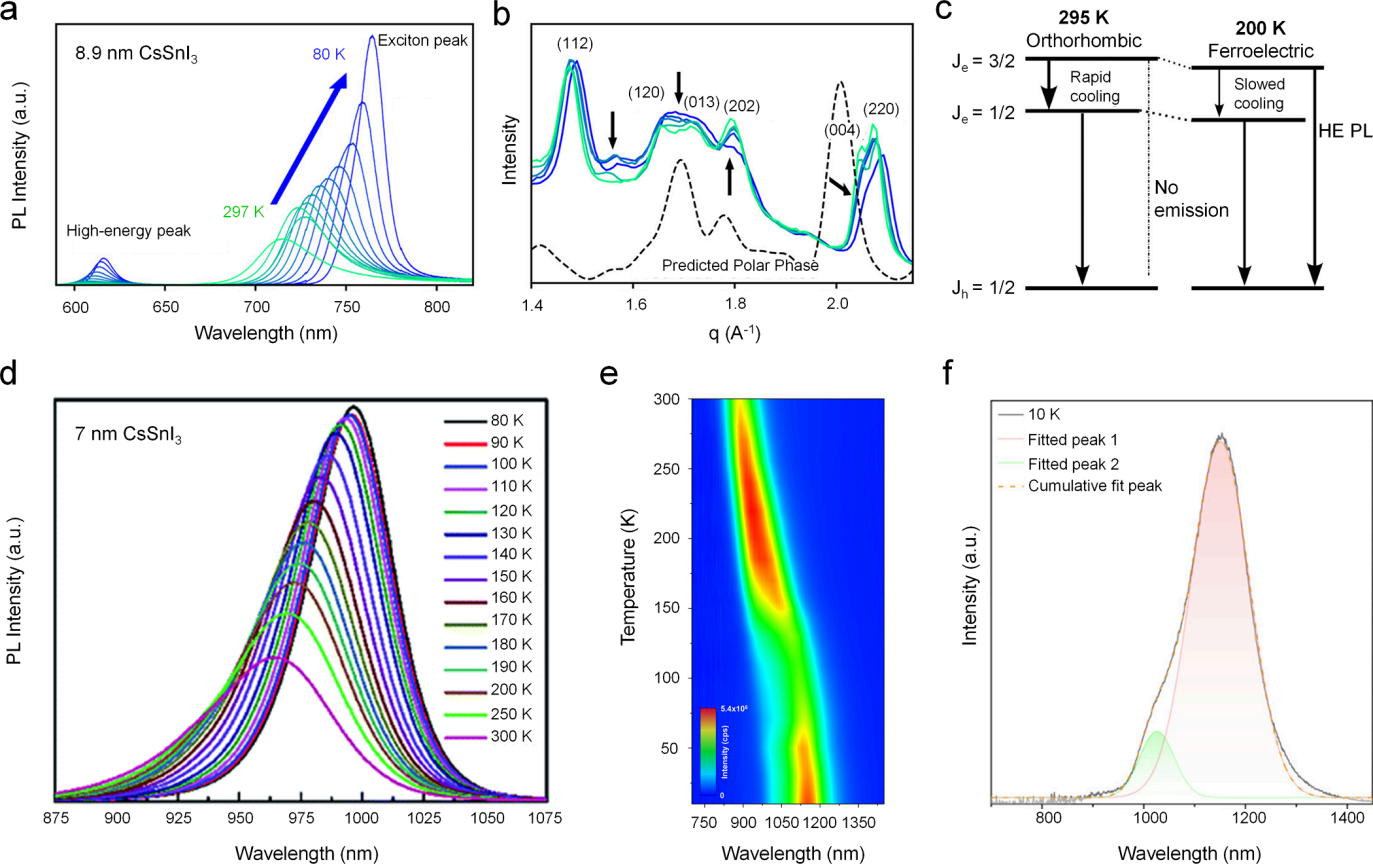
(a) Low-temperature PL spectra and (b) XRD patterns of CsSnI_3_ NC films from 297–80 K. The calculated XRD pattern from a predicted low-temperature polar phase is also displayed in panel (b). (c) Schematic presentation depicting changes in kinetics behavior of bands between 295 and 200 K. Reproduced with permission from ref 36. Copyright 2025 American Chemical Society. (d) Temperature-dependent PL spectra of CsSnI_3_ NCs. Reproduced from ref 42. Copyright 2020 The Royal Society of Chemistry. Licensed under CC BY-NC. (e) Contour plot of the PL spectra upon cooling from 300 to 10 K and (f) PL spectrum and fitted bands taken at 10 K of FASnI_3_ microcrystals synthesized with a 1:3.2 precursor ratio of FA and SnI_2_. Reproduced with permission from ref 58. Copyright 2024 American Chemical Society.

Further insight into this phenomenon was obtained by temperature-dependent XRD analysis.^36^ The diffraction patterns shown in Figure 3b exhibit subtle yet systematic changes, including peak splitting, disappearance of characteristic reflections, and the emergence of a new feature near 1.56 Å^–1^, none of which can be reconciled with the room-temperature orthorhombic phase. Complementary differential scanning calorimetry data revealed multiple thermal events between 270 and 220 K, indicating that the structural reorganization proceeds through a sequence of closely spaced transitions rather than a single abrupt phase change. Based on these observations, the onset of a lower-symmetry polar phase was proposed. Within this phase-transition framework, the unusual PL behavior was rationalized by modifications to the electronic structure. The two emissive features were assigned to transitions involving spin–orbit–split conduction-band manifolds, whose relative energetic positions are depicted schematically in Figure 3c. In the polar phase, the path connecting the upper J = 3/2 and lower J = 1/2 states becomes more tortuous in k-space. The altered curvature introduces a barrier for intervalley relaxation, such that carriers photoexcited into the higher-lying manifold cool less efficiently.

In a seemingly contrasting study by Mahesh et al., 7 nm CsSnI_3_ NCs, with a bandgap of 1.34 eV and room-temperature PL maximum at 938 nm (comparable to that reported by Liu et al.^21^), were employed for temperature-variable PL measurements.^42^ In this case, the PL peak exhibits a monotonic redshift upon cooling from 300 to 80 K (Figure 3d). An exciton binding energy of 55.5 meV was extracted, slightly larger than that observed in CsSnI_3_ polycrystalline films.^59^ Notably, no pronounced excitonic absorption peak was observed for these 7 nm CsSnI_3_ NCs, whereas clear excitonic features were reported for 8.9 nm counterparts. Importantly, both the 7 and 8.9 nm CsSnI_3_ NCs were synthesized in the presence of oleylamine, albeit with different stoichiometries relative to the constituent precursors. The observation of near-band-edge PL from the 7 nm NCs therefore raises questions regarding the assignment of the significantly blueshifted PL observed in the 8.9 nm cousin (Figure 3a). Accordingly, more detailed and carefully controlled investigations are required to ascertain whether the short-wavelength PL indeed originates from the 8.9 nm NCs themselves, or alternatively to rationalize why NCs of this size would emit at such high energies.

Temperature-dependent PL anomalies have also been reported for FASnI_3_ systems. As shown in Figure 3e, Chen et al. reported that the PL maximum of FASnI_3_ microcrystals gradually redshifts from 888 to 1150 nm as the temperature decreases from 300 to 10 K, exhibiting a trend similar to that observed in CsSnI_3_ NCs.^58^ Notably, over a broad range of 110–200 K, FASnI_3_ microcrystals exhibit negative thermal quenching of PL, a phenomenon also observed by Kahmann et al. in FASnI_3_ single crystals.^60^ Upon cooling, suppression of exciton thermal dissociation generally leads to enhanced radiative recombination and increased emission intensity, while structural defects can additionally trap photogenerated carriers that may become thermally activated and contribute to radiative combination. Similar behavior has been observed in other tin halide solids.^61^ Furthermore, below 185 K, a new near-infrared emission band emerges, red-shifting from 926 nm at 185 K to 1026 nm at 10 K (Figure 3f). Chen et al. attributed this emission to defect-related states that become optically active at low temperatures but are quenched at higher temperatures due to phonon-assisted nonradiative recombination. Similar defect-related emissions have been reported in the Ba_1–*x*_Sr_*x*_SnO_3_ series and tentatively assigned to Sn^2+^-related states located approximately 1.4 eV above the valence-band edge.^62^ These observations collectively indicate that structural defects in FASnI_3_ can both induce negative thermal quenching and act as low-temperature emissive centers.

Taken together, the foregoing analyses revealed that a series of seemingly disparate anomalies in ASnI_3_ NCs, spanning size-dependent luminescence, photogenerated charge-carrier dynamics, and temperature-dependent PL behaviors, are in fact closely interconnected. These observations demonstrate that the photophysical properties of ASnI_3_ NCs cannot be interpreted within a single conventional framework, such as quantum confinement alone. Instead, multiple extrinsic and intrinsic factors may act concurrently, obscuring the direct structure–property relationships. In the following sections, we therefore examine several key factors that may give rise to these anomalies, beginning with the role of crystal structure.

## Impact of Crystal Structure on Photophysical Properties

The room-temperature cubic FASnI_3_ phase is commonly treated as a monomorphous structure. Recent evidence suggests that electronic-structure calculations based on such macroscopically averaged monomorphous networks derived from XRD often show substantial deviations from experimental observations, including systematically underestimated bandgaps, dielectric constants dominated by the electronic, negative mixing enthalpy of alloys, and significant deviations from measured pair distribution functions. To address these inconsistencies, Zhao et al. proposed a new concept of “polymorphous networks”, which feature a distribution of low-symmetry local motifs while retaining a high-symmetry average structure (Figure 4a).^63^ Compared with their monomorphous counterparts, polymorphous networks exhibit significantly lower predicted total energies (Figure 4b), larger bandgaps, and dielectric constants dominated by ionic contributions, and show much better agreement with experimentally measured pair distribution functions. The polymorphous nature of cubic perovskites was shown to extend beyond FASnI_3_, encompassing CsSnI_3_ and other oxide perovskites. Given its demonstrated ability to rationalize multiple anomalies in perovskite materials, Dirin et al. adopted this concept to interpret the absorption and PL behaviors of FASnI_3_ NCs.^29^

**Figure 4:**
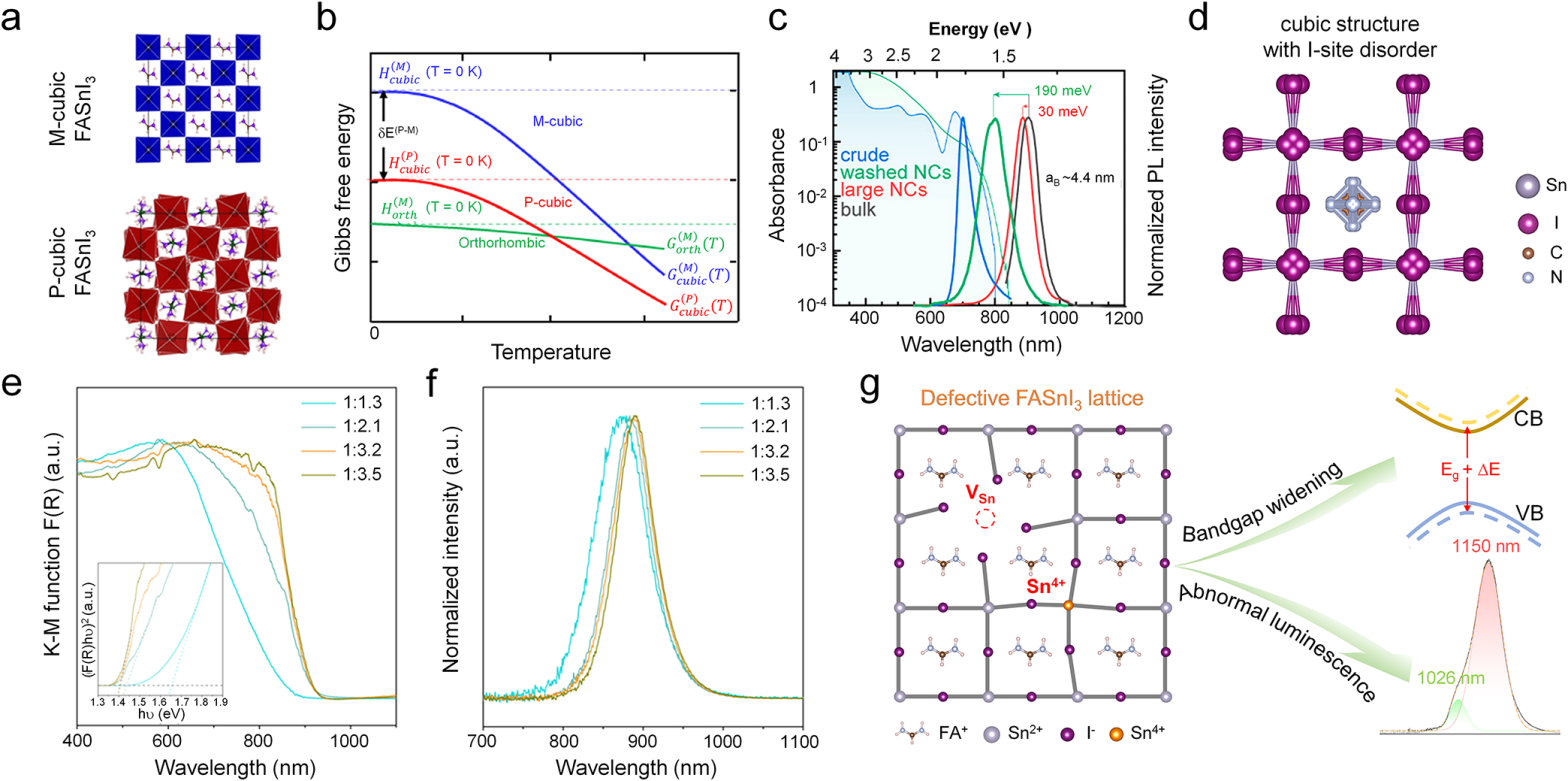
(a) Crystal structures of monomorphous cubic (M-cubic) and polymorphous cubic (P-cubic) phases of FASnI_3_. (b) Schematic representation of the temperature dependence of the enthalpy (H) and Gibbs free energy (G) for the cubic monomorphous phase (blue curves), cubic polymorphous phase (red curves), and the ground-state orthorhombic phase (green curves). The total energy lowering, δE^(P–M)^, of the polymorphous cubic phase relative to the cubic monomorphous phase is also indicated. Reproduced with permission from ref 63. Copyright 2020 American Physical Society. (c) Absorption and PL spectra of colloidal FASnI_3_ NCs with a particle size of ∼10 nm, and PL spectra of bulk FASnI_3_ as well as 200 nm large NCs. (d) Schematic of cubic FASnI_3_ structures with I-site disorder. Reproduced with permission from ref 29. Copyright 2023 American Chemical Society. (e) Absorption spectra of FASnI_3_ microcrystals synthesized under different FA:SnI_2_ precursor ratios. Inset is the Tauc plot for the calculation of the bandgaps. (f) PL spectra of FASnI_3_ microcrystals synthesized with different FA:SnI_2_ precursor ratios. (g) Schematic representation of defect-induced bandgap widening and occurrence of high-energy luminescence at low temperature. Reproduced with permission from ref 58. Copyright 2024 American Chemical Society.

Figure 4c shows the absorption and PL spectra of colloidal FASnI_3_ NCs with a particle size of ∼10 nm, together with PL spectra of bulk FASnI_3_ as well as ∼200 nm particles.^29^ The absorption onset of the ∼10 nm NCs is notably broadened, and the first excitonic peak remains unresolved despite the narrow size dispersion. Furthermore, these NCs exhibit a relatively low intrinsic absorption coefficient of 4 ± 1.7 × 10^3^ cm^–1^, approximately four times lower than that reported for bulk FASnI_3_. In addition, relative to bulk FASnI_3_, the PL peak of the ∼10 nm NCs is blueshifted by ∼190 meV, whereas the 200 nm particles exhibit a much smaller blueshift of ∼30 meV. Such a pronounced modification of the band structure cannot be explained by quantum confinement alone, given that these NCs are ∼10 nm in size, while the exciton Bohr diameter of FASnI_3_ has been estimated to be 7–8.8 nm.^29^ To rationalize these observations, Dirin et al. proposed that the PL blueshift may result from the locally distorted iodide framework in FASnI_3_ NCs, including split iodide positions and bent Sn–I–Sn angles, as evidenced by ^119^Sn NMR measurements (Figure 4d). Such structural distortions can increase the bandgap and reduce the excitonic oscillator strength, resulting in an optical behavior governed primarily by local disorder rather than quantum confinement.

While the concept of polymorphous networks does not account for all reported size–luminescence anomalies in tin-halide perovskite NCs, it nonetheless underscores the importance of careful structural characterization of NCs obtained from different synthetic routes. Such scrutiny is essential for disentangling intrinsic size effects from disorder-induced band-structure modifications and for establishing more reliable structure–PL correlations.

## Impact of Structural Defects and Hole Doping on Photophysical Properties

It is worth noting that most reported ASnI_3_ NCs exhibit extremely low PLQYs, indicating the presence of a high density of structural defects that promote strong nonradiative recombination (Table 2). In this context, Chen et al. performed a detailed analysis of defect-induced bandgap widening in FASnI_3_.^58^ By systematically tuning the FA:SnI_2_ precursor ratios, FASnI_3_ microcrystals with PLQYs ranging from 3.8% to 8.0% were obtained. The sample with the lowest PLQY exhibits a bandgap widening by 0.23 eV relative to microcrystals with higher PLQYs (Figure 4e), accompanied by a pronounced blueshift of the PL peak (Figure 4f). Structural analyses suggested that this bandgap widening originates from defect-induced lattice distortions arising from Sn^4+^ impurities and/or point defects. Notably, such structural defects can also give rise to a new near-infrared PL band at low temperatures (Figure 3f and Figure 4g).

Importantly, the presence of structural defects in ASnI_3_ perovskites is often intrinsically coupled with hole doping. By combining steady-state and time-resolved PL, kinetic carrier-dynamics modeling, and DFT calculations, Treglia et al. established a unified framework linking doping density, trap density, and recombination pathways in FA_0.85_Cs_0.15_SnI_3_.^64^ Pristine films processed from commercial SnI_2_ exhibit high PLQYs (∼20%), a behavior attributed to moderate p-doping. Such doping enhances monomolecular radiative recombination between photoelectrons and dopant-induced holes, thereby partially masking trap-assisted nonradiative losses. In contrast, intentional introduction of small amounts of Sn^4+^ leads to pronounced blueshifts of both absorption and PL spectra, arising from heavier *p*-type doping (Burstein–Moss effect) and/or defect-induced lattice distortions, and is accompanied by substantially reduced carrier lifetimes and PLQYs. However, Milot et al. demonstrated that, at hole densities near 10^20^ cm^–3^, a strong Burstein–Moss effect can increase the absorption onset energy by approximately 300 meV relative to undoped FASnI_3_, without inducing a notable shift in the PL peak.^65^

Taken together, these comparisons underscore that structural defects play a critical role in governing the PL behavior of ASnI_3_ perovskites and that the interplay between structural defects and hole doping should be carefully disentangled to correctly interpret their photophysical properties.

## Impact of 2D Perovskites on Photophysical Properties

The synthesis pathways of tin halide perovskite NCs differ fundamentally from those of their lead analogues because Sn^2+^ exhibits a higher Lewis acidity and greater solubility in polar media.^66^ As a result, Sn^2+^ readily forms complexes with hard donor ligands (R-COO^–^, R-NH_2_), making the 3D ASnI_3_ lattice significantly more susceptible to decomposition into CsI, SnI_2_, and oleates unless the precursor chemistry is carefully controlled.^23^ For this reason, successful syntheses have generally employed both elevated SnI_2_ concentrations and substoichiometric ligand amounts. Although these conditions are essential to stabilize the 3D perovskite phase, excess SnI_2_ simultaneously promotes the formation of 2D RP intermediates, making the ASnI_3_ system chemically more complex than lead halide perovskites.

Gahlot et al. demonstrated that CsSnI_3_ NCs with excitonic features can be obtained only under Sn-rich and ligand-poor conditions.^23^ In this regime, the nucleation pathway proceeds through 2D (R-NH_3_^+^)_2_SnI_4_ sheets, which act as early intermediates and can either convert into 3D CsSnI_3_ NCs or persist to form mixed 3D–2D products, depending on the Cs:Sn precursor ratio. To further map this synthetic mechanistic landscape, the Cs:Sn ratio was varied from 1:6 to 3:1, and the relative formation probabilities of CsSnI_3_ versus competing byproducts were evaluated (Figure 5a-c). In Sn-rich regimes, (Cs:Sn = 1:6, 1:3, 1:2), theoretical models predicted that all Cs^+^ would be incorporated into CsSnI_3_, leaving excess Sn^2+^ and I^–^ in the final reaction mixture. However, this scenario represents an idealized assumption, as the resulting product exhibits a pronounced PL blueshift of ∼0.46 eV relative to bulk CsSnI_3_. When Cs^+^ was introduced in excess, the reaction landscape shifted accordingly. The formation of Cs-oleate, Sn-oleate, and R-NH_3_^+^I^–^ becomes favorable, and layered RP phases, (R-NH_3_^+^)_2_Cs_n–1_Sn_n_I_3n+1_ (*n* > 1), tend to form. At a Cs:Sn ratio of 1:1, this equilibrium breaks down, yielding a broad, red-shifted emission centered at 812 nm (1.53 eV) (Figure 5c). These observations demonstrate that, in the presence of oleylamine, RP-phase formation is closely coupled to precursor stoichiometry and that the coexistence of RP phases with 3D perovskites can substantially influence the observed optical response. As discussed in the “Size–luminescence anomalies” section, more detailed analyses are required to clarify why CsSnI_3_ NCs with sizes of ∼6 and ∼10 nm exhibit PL maxima at ∼600 and ∼711 nm, respectively,^23^ accompanied by notable excitonic absorption features, whereas NCs of comparable sizes reported by other groups display near-band-edge emission at 938 and ∼950 nm with no obvious excitonic absorption.^16,42^

**Figure 5:**
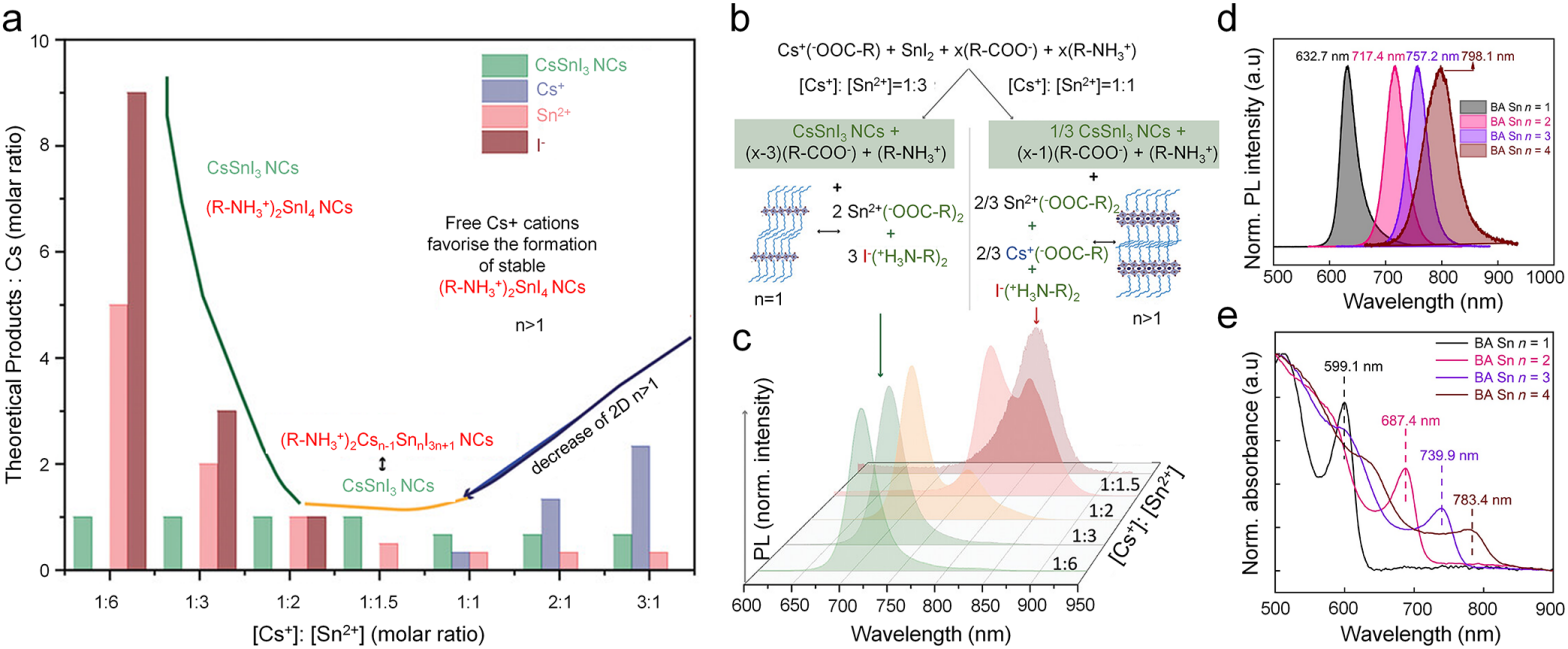
(a) Representation of the theoretical ratio of final products versus Cs as a function of the Cs^+^:Sn^2+^ precursor ratio as calculated from the chemical reaction. (b) Reaction scheme based on experimental results for different ratios of Cs^+^: Sn^2+^. (c) Experimental PL spectra and PL peak evolution for products obtained with different ratios of Cs^+^: Sn^2+^ precursors. Reproduced with permission from ref 23. Copyright 2022 Wiley-VCH. (d) PL and (e) UV–vis absorption spectra of (BA)_2_MA_*n*–1_Sn_*n*_I_3*n*+1_ (*n* = 1–4). Reproduced with permission from ref 67. Copyright 2023 American Association for the Advancement of Science.

To assess the influence of 2D RP impurities on the photophysical behaviors of CsSnI_3_ and FASnI_3_ NCs, a comprehensive survey of reported 2D systems is compiled in Table 3, detailing variations in A-site cations, spacer ligands, and layer thickness (n-value). For a representative system using BA as the spacer ligand and MA as the A-site cation, PL and absorption spectra of RP phases with different layer thickness (n = 1–4) are shown in Figures 5d and 5e, respectively.^67^ Comparison of the optical properties of these 2D phases with those of CsSnI_3_ and FASnI_3_ reveals that, particularly for *n* > 1, their absorption and emission features, typically within the range of 700–800 nm (Table 3), often lie in close proximity to those claimed for 3D NCs (Figure 1; Table 1 and 2). These findings highlight a critical caveat: the potential coexistence of 2D RP byproducts in CsSnI_3_ and FASnI_3_ NC samples can significantly complicate the interpretation of PL measurements, especially given that CsSnI_3_ and FASnI_3_ NCs generally exhibit extremely weak luminescence and low PLQYs, whereas 2D RP phases are comparatively highly luminescent.^68–71^ We therefore emphasize that the widespread use of primary amines, such as oleylamine, as ligands in the synthesis of CsSnI_3_ and FASnI_3_ NCs intrinsically promotes the formation of 2D RP byproducts. Even trace amounts of such phases, which are often too subtle to be detected by conventional XRD or TEM analyses, may nonetheless dominate the observed PL, thereby leading to misassignment of the intrinsic photophysical properties of ASnI_3_ NCs.

**Table 3:** Absorption Onset, PL Maxima, and PLQY for 2D and Quasi-2D Tin Iodide Perovskite Systems with Different A-Site Cations and Spacer Ligands

Perovskite system		Absorption onset (nm)	PL maxima (nm)	PLQY (%)	ref.
*n* = 1	(OLA)_2_SnI_4_ ^a^	604	628.2	0.5	^72^
	(BA)_2_SnI_4_ ^b^	599.1	632.7		^67^
	(PEA)_2_SnI_4_ ^c^	∼605	∼625		^67^
	(2T)_2_SnI_4_ ^d^	∼605	∼625		^67^
	(3T)_2_SnI_4_ ^e^	∼605	∼625		^67^
	(4FPEA)_2_SnI_4_ ^f^		627.2		^73^
	(OLA)_2_SnI_4_	∼600	635		^23^
	PEA_2_SnI_4_	611	623		^74^
	NMA_2_SnI_4_ ^g^	596	616		^74^
*n* = 2	(OLA)_2_[FASnI_3_]SnI_4_	674	689	2.6	^72^
	(BA)_2_[FASnI_3_]SnI_4_	687.4	717.4		^67^
	(PEA)_2_[FASnI_3_]SnI_4_	∼675	∼700		^67^
	(2T)_2_[FASnI_3_]SnI_4_	∼675	∼700		^67^
	(3T)_2_[FASnI_3_]SnI_4_	∼675	∼700		^67^
	(4FPEA)_2_[CsSnI_3_]SnI_4_		697.1		^73^
	(4FPEA)_2_[FASnI_3_]SnI_4_		690.3		^73^
*n* = 3	(BA)_2_[FASnI_3_]_2_SnI_4_	739.9	757.2		^67^
*n* = 4	(BA)_2_[FASnI_3_]_3_SnI_4_	783.4	798.1		^67^

a OLA stands for oleylammonium.

b BA stands for butylammonium.

c PEA stands for phenethylammonium.

d 2T stands for bithiophenylethylammonium.

e 3T stands for 2-([2,2′:5′,2″-terthiophen]-5-yl)­ethan-1-aminium.

f 4FPEA stands for 4-fluoro-phenethylammonium.

g NMA stands for 1-naphthylmethylammonium.

## Perspectives for Future Research

The series of ambiguities observed in the photophysical properties of ASnI_3_ NCs, including anomalies in size- and temperature-dependent PL as well as photogenerated charge-carrier dynamics, cannot be rationalized by a single factor. Although crystal structure, structural defects, hole doping, and 2D RP impurities have been identified as likely contributors, it remains highly challenging to unambiguously correlate a given PL behavior with a specific microscopic origin. To advance toward a more coherent understanding of the observed photophysical properties, we outline below several perspectives for future research.

First, the synthesis of highly luminescent ASnI_3_ NCs, followed by systematic investigation of their structural and photophysical properties, is essential for establishing reliable structure–property correlations. Unlike lead halide perovskites, in which structural defects can be relatively readily suppressed, ASnI_3_ NCs are highly susceptible to both surface and bulk defects.^14^ Photophysical studies conducted on such poorly emissive NCs inevitably conflate defect-mediated effects with intrinsic properties. Encouragingly, recent advances in computationally guided synthesis have rendered the attainment of highly luminescent ASnI_3_ NCs increasingly feasible. For example, theoretical calculations revealed that CsSnI_3_ perovskites exhibit complex defect chemistry: with increasing Sn chemical potential, the formation energies of tin vacancies (V_Sn_), Cs vacancies (V_Cs_), Cs antisite (Cs_Sn_), and interstitial iodide (I_i_) increase, whereas those of iodine vacancies (V_I_) and Sn antisite (Sn_I_) decrease,^21^ indicating that simultaneous suppression of all defect types remains intrinsically difficult (Figure 6a). Further analysis of thermodynamic charge-transition levels revealed that V_Sn_ introduces deep trap states that strongly promote nonradiative recombination, whereas other defects are comparatively shallow (Figure 6b). By fine-tuning reactant ratios under Sn-rich conditions to suppress the most PL-detrimental V_Sn_, CsSnI_3_ NCs with PLQYs of ∼18% have been achieved. In a subsequent study, Chen et al. demonstrated that substantially defect-free NCs cannot be realized solely through chemical-potential control, because Sn-rich conditions preferentially suppress bulk defects, whereas Sn-poor conditions reduce surface defects (Figure 6c, d).^19^ By combining Sn-rich growth conditions with the introduction of exogenous monovalent cations to produce defect-tolerant surfaces, FASnI_3_ NCs with PLQYs as high as 42.4% were obtained. Future efforts along this direction, coupled with precise control over nucleation and growth, may yield NCs with PLQYs approaching those of their Pb-based counterparts.

**Figure 6:**
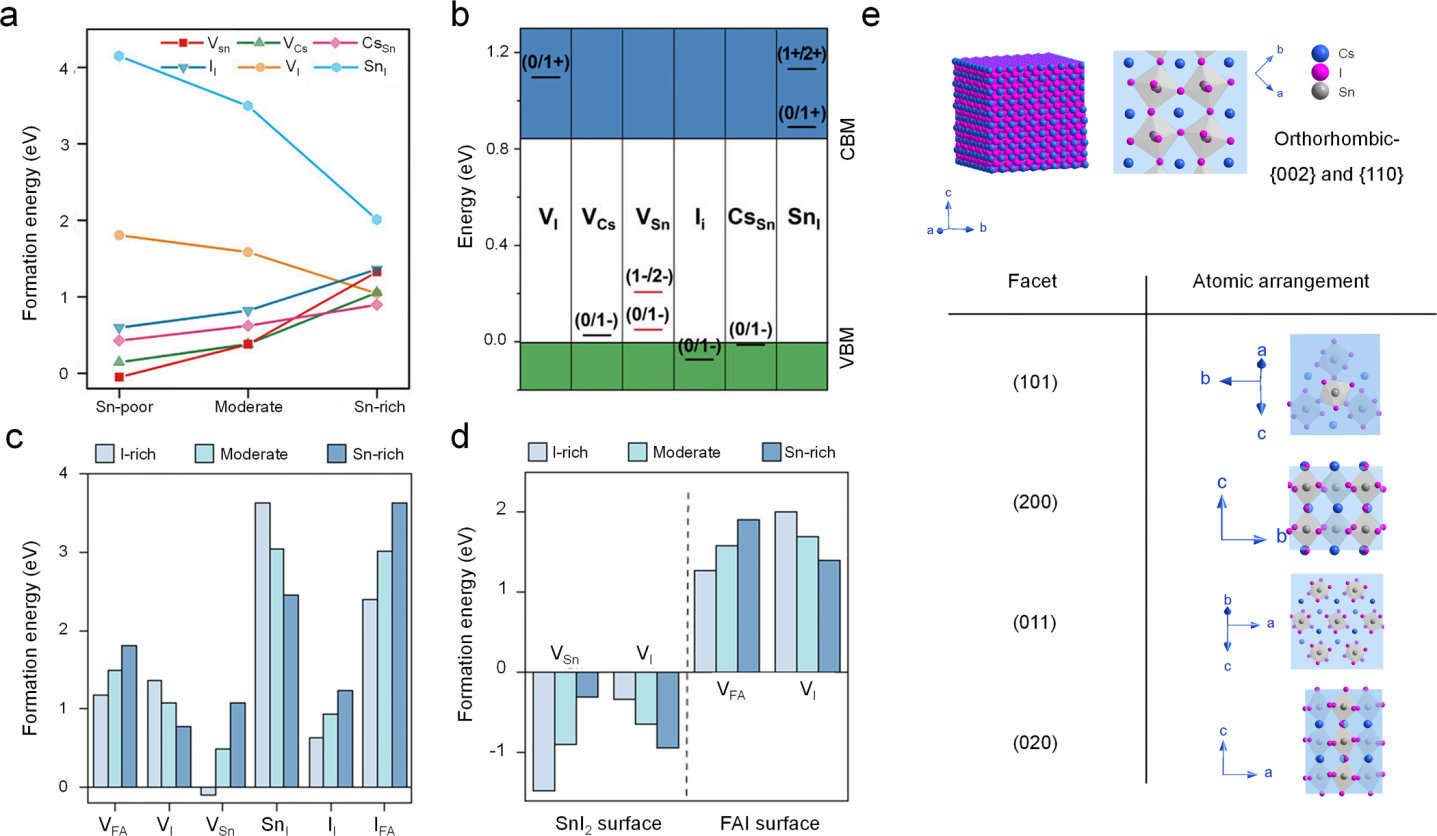
(a) Calculated defect formation energies for 2 × 2 × 2 orthorhombic CsSnI_3_ supercells at Sn-poor/I-rich, moderate, and Sn-rich/I-poor conditions. (b) Defect charge-transition levels of CsSnI_3_. Reproduced with permission from ref 21. Copyright 2021 American Chemical Society. (c) Formation energies for various point defects within the bulk crystal structure under tin-rich, moderate and iodine-rich conditions and (d) surface defect formation energies for V_Sn_ and V_I_ on the SnI_2_ surface and V_FA_ and V_I_ on the FAI surface in FASnI_3_. Reproduced with permission from ref 19. Copyright 2025 Nature Publishing Group. (e) Schematic presentation of atomic arrangement in different facets of CsSnI_3_ crystals. All models were designed using Diamond 4.0 with the CIF file code no. 4117959.

Second, achieving phase-pure ASnI_3_ NCs is equally critical for the reliable interpretation of photophysical properties, as the unintended formation of impurity phases can lead to misleading structural and optical assignments. Although a wide variety of ligand systems have been explored for lead halide perovskites,^1,75,76^ the synthesis of ASnI_3_ NCs still predominantly relies on combinations of alkyl amines and acids (e.g., oleylamine and oleic acid) (Table 1). Considering that oleylamine is particularly prone to promoting the formation of 2D RP phases under Sn- and halide-rich conditions, exploration of alternative ligand chemistries, including zwitterionic ligands,^19^ secondary or tertiary alkylamines, and phosphorus- or sulfur-based ligands, may favor formation of the desired 3D phase while suppressing 2D phases. As an example, Chen et al. recently demonstrated that the zwitterionic ligand, dioleoyl-*sn*-glycero-3-phosphocholine, enables the synthesis of FASnI_3_ NCs without detectable 2D phase impurities.^19^ Moreover, rational ligand design may simultaneously improve phase purity and enhance NC stability, thereby providing a more robust platform for probing the intrinsic photophysics of ASnI_3_ NCs.

Third, dopant engineering, which has been widely exploited in lead halide perovskite NCs, represents another promising strategy for obtaining high-quality ASnI_3_ NCs.^77,78^ Theoretical studies predicted that substitutional divalent doping at the B site of CsSnI_3_ with elements such as Co, Cu, and Zn stabilizes the photoactive black phase that is energetically favored over the yellow phase,^79^ while dopants such as Y, Sc, Al, Zr, Nb, Ba, and Sr can pin the Fermi level deeper within the bandgap and suppress *p*-type self-doping.^80,81^ Despite these predictions, experimental attempts to dope ASnI_3_ NCs and bulk phases with elements such as Sb, Bi, or Ge have thus far yielded only modest improvements in PLQY and stability, falling short of practical requirements.^14,82,83^ This discrepancy highlights the need for new synthetic strategies that enable effective dopant incorporation while simultaneously minimizing defect formation. Progress in this area could unlock access to high-quality ASnI_3_ NCs suitable for rigorous photophysical investigations.

Fourth, control over NC morphology and facet exposure, which has advanced substantially for lead halide perovskite NCs over the past decade,^84–88^ remains comparatively underexplored for ASnI_3_ systems. Although progress has been made in defect management, PLQY enhancement, and size control, most reported ASnI_3_ NCs still predominantly adopt cubic or platelet-like morphologies, exposing crystallographically equivalent facets with identical atomic arrangements (e.g., (002) or (110) facets in the orthorhombic phase and (100) facets in the cubic phase). To achieve a more complete understanding of intrinsic photophysics, it is imperative to move beyond this restricted morphological space. As illustrated in Figure 6e, distinct crystallographic facets of CsSnI_3_ exhibit different atomic configurations and surface terminations, which are expected to influence defect formation and surface chemistry in facet-specific ways. Access to NCs exposing a broader range of facets may therefore reveal new optical signatures and, importantly, provide mechanistic insight into crystal degradation pathways, which are strongly governed by surface structure and remain a central challenge for tin halide perovskites.

In parallel with anticipated synthetic advances, rigorous characterization with strong discriminative power is indispensable for establishing reliable structure–property relationships in ASnI_3_ NCs. Beyond conventional techniques such as laboratory XRD and TEM, advanced characterization tools capable of resolving minor, spatially localized, or weakly emissive secondary phases, such as scanning TEM–cathodoluminescence, synchrotron-based XRD, and total scattering/pair distribution function analyses, should be systematically employed, particularly given the pronounced propensity of ASnI_3_ systems to form trace 2D perovskite phases. Equally critical is comprehensive and carefully designed photophysical characterization. We emphasize that temperature-dependent PL measurements spanning a broad spectral window from the visible to the near-infrared should be routinely conducted, especially when the observed emission energies deviate markedly from their bulk counterparts. Such measurements are particularly crucial in cases where ASnI_3_ NCs exhibit weak or negligible room-temperature emission, while coexisting impurity phases are highly luminescent, a scenario that can readily lead to erroneous assignment of emission origins.

Overall, although investigations into the optical properties of ASnI_3_ NCs remain at an early stage and a broad range of anomalous photophysical behaviors have been reported, a rigorous understanding of their structure–property relationships is well within reach, provided that studies are conducted on phase-pure, highly luminescent, and carefully characterized systems. We therefore underscore that exceptional care must be exercised when interpreting seemingly anomalous optical phenomena, as such observations do not necessarily reflect the intrinsic photophysics of ASnI_3_ NCs but may instead originate from extrinsic contributions, including localized structural disorder, structural defects, p-type doping, trace 2D impurity phases, or combinations thereof. With sustained efforts in this direction, the weak-, intermediate-, and strong-confinement regimes, well established in lead halide perovskite NCs,^1,89^ should become accessible in ASnI_3_ systems. Importantly, the considerations and methodological strategies articulated in this Perspective extend beyond iodide-based compositions and are equally applicable to bromide and chloride cousins, for which fundamental structure–photophysics correlations remain comparably underdeveloped and warrant systematic investigation.
